# Risk factors for cancer development in type 2 diabetes: A retrospective case-control study

**DOI:** 10.1186/s12885-016-2836-6

**Published:** 2016-10-10

**Authors:** Mariusz Dąbrowski, Elektra Szymańska-Garbacz, Zofia Miszczyszyn, Tadeusz Dereziński, Leszek Czupryniak

**Affiliations:** 1 Faculty of Medicine, Institute of Nursing and Health Sciences, University of Rzeszow, Al. Mjr. W. Kopisto 2a, 35-310 Rzeszów, Poland; 2 Department of Infectious and Liver Diseases, Medical University of Łódź, ul. Kniaziewicza 1/5, 91-347 Łódź, Poland; 3 Private Clinic of Internal Diseases and Diabetes, ul. 3 Maja 18, 37-700 Przemyśl, Poland; 4 NZOZ Esculap, ul. Dworcowa 8, 88-140 Gniewkowo, Poland; 5 Department of Internal Diseases and Diabetology, Warsaw Medical University, ul. S. Banacha 1a, 02-097 Warsaw, Poland; 6 NZOZ “Beta-Med”, Plac Wolności 17, 35-073 Rzeszow, Poland

**Keywords:** Cancer, Diabetes, Insulin, Metformin, Obesity

## Abstract

**Background:**

The risk of several types of cancer is increased in type 2 diabetes mellitus. The earliest possible diagnosis of cancer – difficult within regular outpatient diabetes care - is of utmost importance for patients’ survival. The aim of this multicenter, retrospective (years 1998–2015), case-control study was to identify risk factors associated with malignancy in subjects with diabetes treated in a typical outpatient setting.

**Methods:**

In the databases of 3 diabetic and 1 primary care clinics 203 patients (115 women) with type 2 diabetes mellitus who developed malignancy while treated for diabetes were identified. The control group consisted of 203 strictly age- and gender matched subjects with type 2 diabetes without cancer. Factors associated with diabetes: disease duration, antidiabetic medications use and metabolic control of diabetes were analyzed. Also other variables: BMI (body mass index), smoking habits, place of residence and comorbidities were included into analysis.

**Results:**

The most prevalent malignancies in men and women together were breast cancer (20.7 %) and colorectal cancer (16.3 %). HbA_1c_ (hemoglobin A_1c_) level ≥8.5 %, obesity and insulin treatment in dose-dependent and time-varying manner demonstrated significant association with increased risk of malignancy, while metformin use was associated with a lower risk of cancer. Diabetes duration, comorbidities, smoking habits, place of residence and aspirin use did not show significant association with risk of malignancy.

**Conclusions:**

In the outpatient setting the obese patients with poorly controlled insulin treated type 2 diabetes mellitus should be rigorously assessed towards malignancies, particularly breast cancer in women and colorectal cancer in men.

## Background

Association between diabetes and cancer has been known for decades [1, 2]. Type 2 diabetes mellitus (T2DM) can be considered as a risk factor of several types of malignant neoplasms [3–11]. In cancer development both genetic and environmental factors play an important role [12, 13]. Among possible biological mechanisms directly linking diabetes and cancer, hyperinsulinemia, hyperglycemia and inflammation are pointed out [14–16].

It is widely assumed that glucose-lowering therapy may influence malignancy risk in diabetic subjects. Metformin is considered to play a protective role in cancer development and outcomes [17], whilst exogenous insulin use seems to be associated with an elevated cancer risk [18]. Oncogenic effects of newer antidiabetic medications is still a matter of uncertainty [19]. Since the earliest possible diagnosis of cancer is of utmost importance for patients’ survival, identification of clinically relevant risk factors of cancer among diabetic patients may be helpful in identifying subjects at greater risk of malignancy.

The aim of this multicenter, retrospective, case-control study was to identify risk factors associated with malignancy in subjects with diabetes treated in a typical outpatient setting.

## Methods

The study was approved by the institutional Bioethics Committee at the University of Rzeszow and by the all appropriate administrative bodies. The study was carried out in accordance with ethical standards laid down in Polish regulations and in an appropriate version of the Declaration of Helsinki (as revised in Brazil 2013).

After Bioethics Committee approval, we performed retrospective analysis of existing individual patients’ records in the databases of 3 diabetic and 1 primary care clinics. Inclusion criteria for the ‘case’ group included: cancer diagnosed after diagnosis of type 2 diabetes, at least one HbA_1c_ measurement before or at the time of cancer diagnosis, date of diabetes diagnosis, diabetes treatment, BMI and history of comorbidities available. We identified 203 patients (115 women) with T2DM eligible for analysis. Data analysis covered the period from January 1998 (the first eligible patient with diagnosed cancer) to 30 April 2015. The mean age of diabetic patients at the time of cancer diagnosis was 67.1 ± 9.7 years, and 141 persons were aged ≥65 years. The control group consisted of 203 strictly age- and gender matched subjects with T2DM without cancer. Patients were selected from the same databases in the case-control manner, with the 1:1 ratio. For each ‘case’ patient, an eligible ‘control’ subject with the same gender, and with the nearest possible date of birth was chosen, and any given pair was recruited always at one center to avoid impact of different treatment algorithms used in different clinics. Individuals who died within the analyzed time period but before the moment of data collection were also included into the analysis if their data were available. Data for patients with malignancy were taken from the period preceding cancer diagnosis (index time). Data for the ‘control’ subjects were assessed from the same index time, i.e., if the ‘case’ patient had cancer diagnosed in April 2009, the data for his/her comparator were taken from the same period.

In both groups metabolic control of diabetes (mean HbA_1c_ from the preceding up to 3 years before index time, if available), diabetes duration, antidiabetic medications (also from the preceding up to 3 years, and each drug was classified as “used” if it was taken for at least 6 months), mean insulin dose from the preceding 6 months, and duration of insulin treatment up to the moment of cancer diagnosis were analyzed. Also place of residence (rural, small cities or urban), smoking habits (current, former or never smoker), presence of comorbidities (hypertension, hyperlipidemia and cardiovascular disease), BMI and use of aspirin were also included into the analysis. All included patients were of Caucasian ethnicity. Detailed characteristics of both groups is presented in the Table 1.

**Table 1:** Characteristics of the case and control groups

Parameter	Malignancy	No malignancy	*P* value*
Age at index time (years) (mean ± SD)	67.1 ± 9.7	67.1 ± 9.7	-
< 65 years (n)	62 (30.5 %)	62 (30.5 %)	
≥ 65 years (n)	141 (69.5 %)	141 (69.5 %)	
Gender			-
male (n)	88 (43.3 %)	88 (43.3 %)	
female (n)	115 (56.7 %)	115 (56.7 %)	
Place of residence			0.264
rural (n)	38 (18.7 %)	44 (21.7 %)	
cities <50,000 inhabitants (n)	27 (13.3 %)	36 (17.7 %)	
cities >50,000 inhabitants (n)	138 (68.0 %)	123 (60.6 %)	
Smoking habits			0.838
never smokers (n)	115 (56.7 %)	117 (57.6 %)	
former smokers (n)	54 (26.6 %)	57 (28.1 %)	
current smokers (n)	33 (16.3 %)	29 (14.3 %)	
unknown status (n)	1 (0.5 %)	-	
BMI (kg/m^2^) (mean ± SD)	30.8 ± 5.3	30.1 ± 4.7	0.103
Comorbidities			
cardiovascular disease (n)	50 (24.6 %)	58 (28.6 %)	0.369
hypertension (n)	177 (87.2 %)	179 (88.2 %)	0.763
hyperlipidemia (n)	153 (75.4 %)	159 (78.3 %)	0.480
Diabetes duration (years) (mean ± SD)	10.7 ± 7.4	10.3 ± 8.1	0.262
HbA_1c_ (%) (mean ± SD)	7.39 ± 1.21 %	7.30 ± 1.06 %	0.755
Antidiabetic medications			
metformin (n)	126 (62.1 %)	167 (82.3 %)	<0.001
sulfonylurea (n)	83 (40.9 %)	101 (49.8 %)	0.090
acarbose (n)	18 (8.9 %)	14 (6.9 %)	0.581
DPP-4 inhibitor (n)	11 (5.4 %)	6 (3.0 %)	0.322
insulin (n)	110 (54.2 %)	81 (39.9 %)	0.005
insulin dose (IU/kg/24 h) (mean ± SD)	0.59 ± 0.31	0.53 ± 0.24	0.407
insulin duration (years) (mean ± SD)	6.2 ± 5.6	6.5 ± 4.9	0.529
Aspirin use (n)	102 (52.6 %)	102 (52.8 %)	0.957
unknown status	9	10	

*NS* non significant

* between malignancy and non-malignancy groups

Current place of residence was taken into analysis with the exception of patients, who moved in the last year. In such cases a previous place of residence was taken into account. Patients were considered as a current, former or never smokers according to definition stated by Centers for Disease Control and Prevention [20]. Hypertension was considered if blood pressure values were ≥140 mmHg for systolic, and/or ≥90 mmHg for diastolic blood pressure, or if antihypertensive medications were used. Hyperlipidemia was recognized if LDL-cholesterol level was ≥2.6 mmol/L (100 mg/dl) and/or triglycerides concentration was ≥1.7 mmol/L (150 mg/dl), or hypolipemic drugs were used. Cardiovascular disease was confirmed if the patient had a history of non-fatal myocardial infarction, hospitalization for acute coronary syndrome, non-fatal stroke, revascularization or amputation.

Statistical analysis of the data was performed using SigmaPlot for Windows version 12.5 (Systat Software Inc., San Jose, CA, USA). The analysis was performed in 2 stages. In the first stage comparison of the two groups was made. The continuous data were analyzed using an unpaired two-tailed Student’s *t*-test or by a Mann-Whitney rank sum test where appropriate. The categorical data were compared using *χ* ^2^ test. In the second stage patients were divided into subgroups according to BMI (<25.0, 25.0–29.9, 30.0–34.9 and ≥35.0 kg/m^2^), diabetes duration (<5.0, 5.0–9.9, 10.0–14.9 and ≥15.0 years), insulin dose (no insulin, <0.50 and ≥0.50 IU/kg) and duration of insulin treatment (no insulin, <5.0, 5.0–9.9 and ≥10.0 years). For the assessment of the effect of treatment or analyzed risk factors on cancer occurrence OR (odds ratios) and 95 % CI (confidence intervals) were calculated in univariate and in multiple logistic regression models. A *P* value <0.05 was considered statistically significant.

## Results

**Fig. 1:**
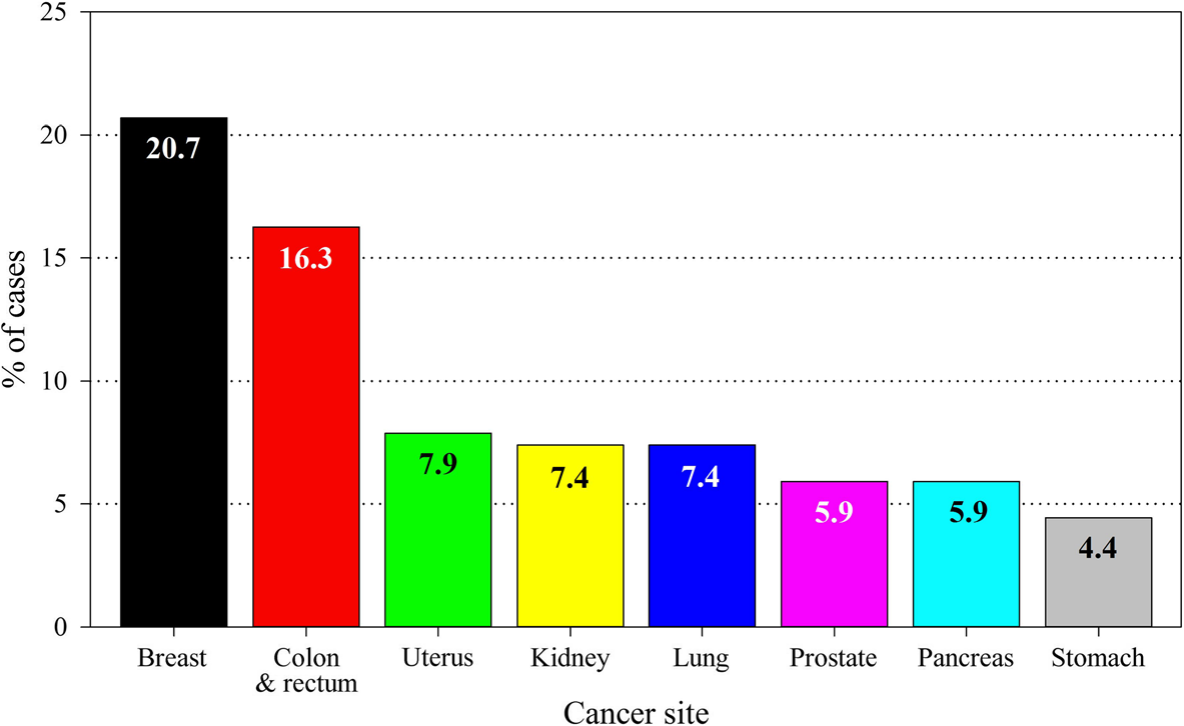
Cancer sites with over 4 % prevalence in the studied population

The most prevalent malignancies in the whole group were: breast, colorectal and uterine cancers (Fig. 1). Among women the most prevalent cancer sites were: breast (36.5 %), uterus (13.9 %), colon with rectum (9.6 %), lung (5.2 %) and stomach (4,4 %), while in men there were: colon with rectum (25.0 %), prostate (13.6 %), kidney (10.2 %), lung (10.2 %) and pancreas (9.1 %).

### Metabolic control

**Fig. 2:**
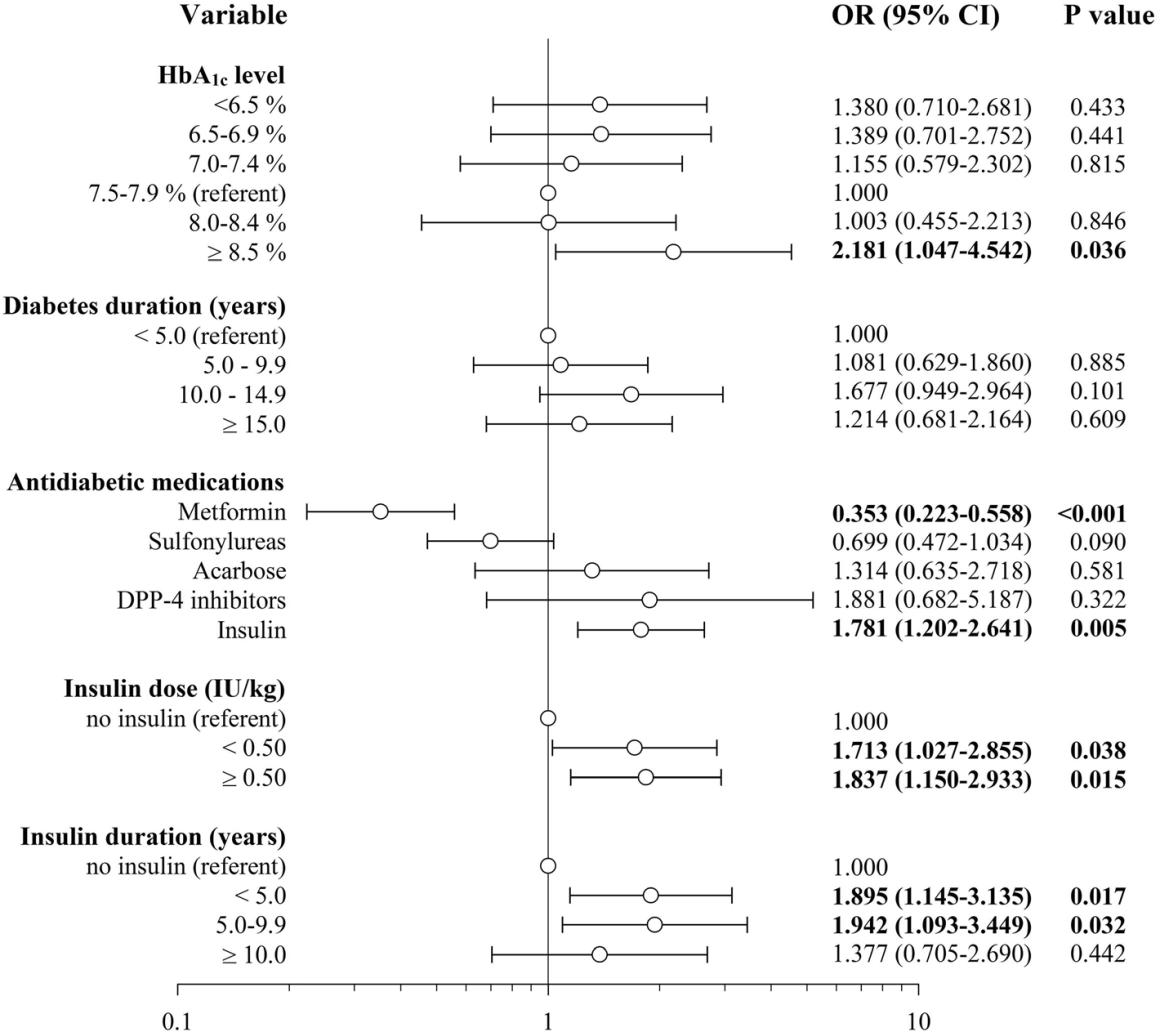
Diabetes-related risk factors of cancer occurrence

Mean HbA_1c_ level was not significantly different between the case and control groups. However, a sharp increase of cancer risk was observed among patients with HbA_1c_ level ≥8.5 % (Fig. 2), and these patients had significantly elevated risk of malignancy compared with the remaining subjects, OR 1.802 (1.030–3.153), *p* = 0.037.

### Diabetes duration

Duration of diabetes did not differ significantly between the two groups. Also when patients were divided into four groups according to diabetes duration, no significant differences were found (Fig. 2).

### Diabetes treatment

Significantly fewer patients in the case group were treated with metformin compared to the control group (Table 1). This difference was significant both in the univariate analysis and after adjustment for BMI, diabetes duration, metabolic control, other antidiabetic medications use and all other variables. Insulin use both in the crude analysis and after adjustment for BMI, diabetes duration and metabolic control was associated with significantly elevated risk of cancer occurrence. After adjustment for other variables in the multiple logistic regression analysis it was attenuated to non-significant level. No significant associations were found for other antidiabetic medications, with the exception of DPP-4 (dipeptidyl-peptidase −4) inhibitors, which after adjustment to all analyzed covariates appeared to be associated with elevated risk of cancer occurrence (Fig. 2 and Table 2). However, only 17 patients (4.2 %) among the whole group of 406 subjects were treated with these medications.

**Table 2:** Adjusted risk of malignancy associated with antidiabetic medications, use vs. non-use

Antidiabetic medication	OR^a^ (95 % CI)	*P* value	OR^b^ (95 % CI)	*P* value	OR^c^ (95 % CI)	*P* value
Metformin	**0.294** **(0.182–0.478)**	**<0.001**	**0.318** **(0.193–0.523)**	**<0.001**	**0.310** **(0.183–0.525)**	**<0.001**
Sulfonylurea	0.735 (0.493–1.094)	0.129	0.859 (0.547–1.349)	0.508	0.906 (0.563–1.456)	0.683
Acarbose	1.333 (0.640–2.776)	0.443	1.372 (0.635–2.967)	0.421	1.245 (0.564–2.747)	0.587
DPP-4 inhibitors	1.954 (0.698–5.468)	0.202	2.809 (0.947–8.331)	0.063	**3.468** **(1.082–11.112)**	**0.036**
Insulin	**1.964** **(1.227–3.144)**	**0.005**	1.509 (0.879–2.588)	0.135	1.735 (0.986–3.053)	0.056

^a^ adjusted for BMI, diabetes duration and metabolic control

^b^ adjusted for BMI, diabetes duration, metabolic control and antidiabetic medications use

^c^ adjusted for BMI, diabetes duration, metabolic control, antidiabetic medications use, smoking history, place of residence, presence of comorbidities and aspirin use

Data presented in bold are statistically significant

Although mean insulin dose and mean duration of insulin use were not significantly different between the case and control groups, insulin treatment has shown association with the risk of malignancy occurrence in a dose-dependent and time-varying manner (P_trend_ 0.015 and 0.027 respectively). The risk of cancer was increasing together with increasing insulin dose and the highest risk was revealed in patients using insulin at a dose ≥0.50 IU/kg. Interestingly, the highest risk was observed in the first 10 years of insulin therapy and it was decreasing thereafter (Fig. 2).

### BMI

**Fig. 3:**
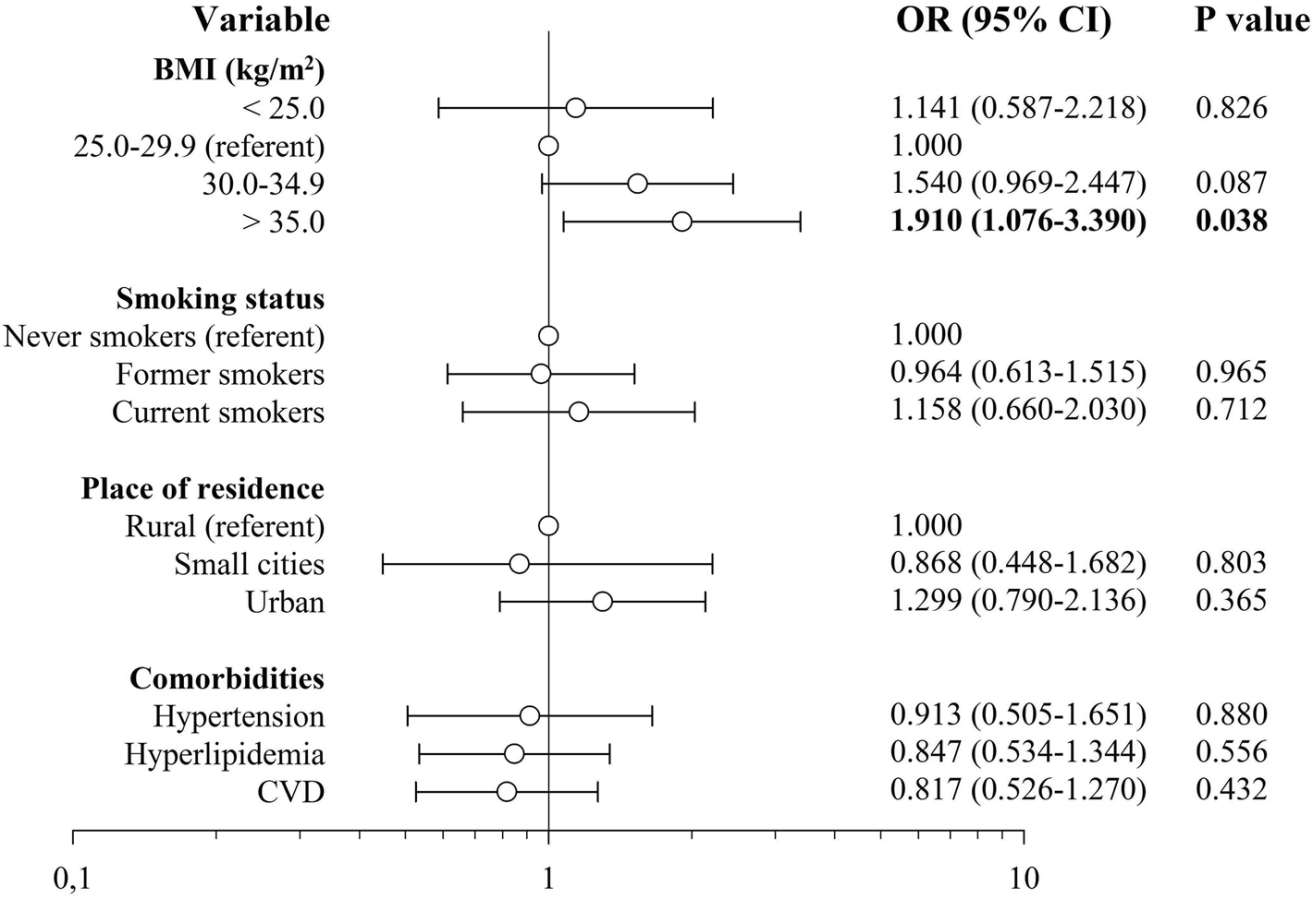
Anthropometric and demographic-related risk factors of cancer occurrence

Mean BMI was not significantly different between the case and control groups. However, risk of malignancy was increasing with the increasing BMI (Fig. 3). Obesity (BMI ≥30 kg/m^2^) was associated with significantly elevated risk of malignancy, OR 1.608 (95 % CI 1.087–2.380), P = 0.022 compared to patients with lower BMI. After adjustment for duration of diabetes, its metabolic control and antidiabetic medications use this relationship become even stronger, OR 2.013 (1.310–3.092), *P* = 0.001.

### Other variables

None of the other analyzed variables had significant effect on risk of malignancy among study participants (Fig. 3). Only among patients with lung cancer smoking (ever vs. never) was associated with significantly elevated risk of malignancy, OR 11.000 (1.998–60.572), P = 0.003. No association was found for other site-specific cancers.

## Discussion

Although the link between diabetes and malignant neoplasms is well known and many site-specific cancers are more prevalent in diabetic patients [3–11], the exact risk factors of cancer in diabetic population have not been fully determined. Also in our study prevalence of breast, colorectal, uterine, kidney, pancreatic and gastric cancers among diabetic patients was higher, while proportion of patients with prostatic and lung cancers was lower than observed in the general Polish population [21], which is in line with majority of other observations [4–7, 22].

Our retrospective, multicenter, case-control study revealed that the following diabetes-related factors may be associated with cancer occurrence: poor metabolic control, obesity and antidiabetic medications use. Importantly, age and gender were not included into risk analysis, because the patients were strictly matched according to them.

Data regarding association between HbA_1c_ level and risk of malignancy are divergent. Some studies showed continuous relationship between increasing HbA_1c_ level and cancer risk [23–25], others found non-linear association [26, 27] (the second one only among women), or elevated risk of malignancy above the specified HbA_1c_ threshold of 7.5 % (colorectal cancer) [28]. In the study by Miao Jonasson et al. no relationship between HbA_1c_ level and cancer was found [29]. In the recent meta-analysis significant association between chronic hyperglycemia and elevated risk of several types of malignancies with the exception of prostate cancer was demonstrated [30]. We took to the analysis the mean HbA_1c_ level from a longer period of time preceding cancer diagnosis considering it as better reflecting the overall exposure to glucose than single measurement. In our study risk of malignancy was rising rapidly at the HbA_1c_ level equal or above 8.5 %. de Beer and Liebenberg found similar HbA_1c_ threshold for the risk of colorectal and breast cancers [30]. In our study these cancers were 37 % of all malignancies, which partly explains our findings.

Some of the studies cited above were performed in diabetic populations [23–25, 29], while other were conducted in both non-diabetic and diabetic subjects [26–28]. Although deleterious effect of glucose and elevated cancer risk as a function of HbA_1c_ may be seen also in a higher versus lower values within a normal range, it can be more pronounced at high glucose concentrations. The higher threshold found in our study can be explained by the fact that prolonged hyperglycemia leads to formation of ROS (reactive oxygen species) and to accumulation of AGEs (advanced glycation end products). AGEs stimulate their specific receptor RAGE (receptor for AGE) which leads to increased inflammation through the activation of the nuclear transcription factor NF-κB and formation of ROS in the cells, which have mutagenic effect and cause DNA damage. This pathway is considered to play an important role in both inflammation and carcinogenesis [31, 32]. Chronic hyperglycemia activates these biological processes and thus a higher HbA_1c_ value may reflect higher cancer risk in poorly controlled diabetes. In addition, glucose serves is a primary energy source for cancer cells, and higher glucose concentrations may accelerate cancer growth [33, 34].

Data regarding effect of diabetes duration on cancer risk are scarce. Johnson et al. and the Danish registry study documented highest cancer risk occurring immediately after diabetes diagnosis [35, 36]. However, Li et al. demonstrated opposite results, with the lowest risk of malignancy in the first 5 years from the onset of diabetes, and the highest cancer risk among patients with diabetes lasting over 15 years [37]. In our study there was a clear tendency towards lowest cancer risk in the first years, and the highest risk between 10 and 15 years after diagnosis of diabetes. The increasing risk of cancer incidence with duration of diabetes can be explained by cumulative effect of hyperglycemia, use of insulin, and weight gain developing in the course of the disease. In addition, increasing age itself is strongly associated with increasing cancer risk both in diabetic and non-diabetic population [21, 38].

Impact of antidiabetic medications on cancer risk has been widely discussed in recent years. As a progressive disease type 2 diabetes requires intensification of treatment over time, from lifestyle modification through oral therapy in different regimens, to insulin treatment. Thus, a clear impact of antidiabetic medications on cancer risk is difficult to determine.

Our study demonstrated highly significant reduction of cancer risk among metformin users, which is in line with many [17, 19, 39, 40] but not all [41] studies. The mechanisms of the anti-cancer effect of metformin include inhibition of cancer cells growth through stimulation of AMPK (AMP-activated protein kinase) and its regulator LKB1 (liver kinase B1), which is known to act as a tumor suppressor protein. In addition, metformin also directly inhibits mTOR (mammalian target of rapamycin) pathway [42] and may have a role of immunomodulator [43].

Sulfonylurea (SU) use in our study did not show relationship with cancer risk. Data from other observations are divergent. Soranna et al. demonstrated neutral effect of SU derivates on the risk of malignancy [39]. This was confirmed by Monami et al. with the exception of gliclazide, which appeared to be protective [44]. On the other hand, recently Thakkar et al. revealed increased cancer risk among SU users [40].

Current evidence from observational studies indicate harmful effect of insulin on the cancer risk [18, 37]. In our study insulin use was associated with a dose-dependent elevated risk of malignancy. Similar relationship was also observed by Holden et al. [45]. Regarding duration of insulin treatment, risk of malignancy in our observation was highest in the first 10 years of treatment, and become insignificant with a longer insulin use. This phenomenon can be explained by increased cancer and also coronary heart disease mortality observed among diabetic patients treated with insulin [46]. Other studies showed increased risk of malignancy associated with insulin use after 4 years of insulin treatment [18]. In general, insulin is a potent growth stimulating hormone acting through insulin and IGF-1 (insulin-like growth factor-1) receptors [34, 47, 48]. On the other hand, landmark prospective studies in type 2 diabetes did not confirm elevated risk of malignancy associated with more intensified treatment [49]. Also Outcome Reduction with an Initial Glargine Intervention (ORIGIN) trial did not demonstrated raised cancer risk among insulin users [50]. However, these studies were of limited duration, also relatively low doses of insulin were used in the ORIGIN trial.

The number of patients treated with other antidiabetic medications in our study was small, therefore we were unable to determine their relationship with cancer risk. Acarbose (the α-glucosidase inhibitor) is not popular due to its well-known side effects. DPP-4 inhibitors are not reimbursed in Poland and their utilization is low. In our study, after adjustment to all analyzed variables they demonstrated significant association with cancer risk. However, it is worth to notice that only 17 patients were treated with these agents and in this case random effect cannot be excluded. Pioglitazone and SGLT-2 inhibitors are also not reimbursed and, in addition, pioglitazone was not available on market in Poland up to 2014, thus number of patients using these medications is extremely low.

Obesity is a well-recognized risk factor of several types of cancer [51, 52]. Our study confirmed association between obesity and risk of malignancy in diabetic population. Obese patients, especially with severe obesity (BMI ≥35 kg/m^2^), had significantly higher risk of cancer occurrence compared with non-obese subjects. It should be remembered that with cancer development body weight frequently decreases, the fact which may mar analysis of the results. Insulin resistance, hyperinsulinemia, elevated levels of IGF-1, inflammation, increased sex hormones bioavailability and hyperglycemia are considered to be responsible for increased cancer risk in obese individuals [53].

Smoking is known to be associated with elevated risk of several site-specific cancers, especially lung cancer [54]. Interestingly, in our study number of never, former and current smokers was not significantly different in case and control groups, and smoking was not associated with elevated overall cancer risk. However, not surprisingly, number of current and former smokers was significantly higher in patients with lung cancer related to their comparators.

For other analyzed variables, including place of residence, presence of comorbidities and aspirin use relationship with risk of malignancy was not revealed.

The limitations of our study include its retrospective and observational design, and relatively small sample size which has influenced the statistical power of our findings, and has not allowed to demonstrate other possible relationships for which positive trends were observed. Also immortal time, time-window and time-lag biases despite our best efforts cannot be excluded [55]. Another limitation is low number of users of oral drugs other than sulfonylurea derivatives and metformin. In addition, all main cancer risk factors confirmed in the study e.g., insulin use, HbA_1c_ level and obesity heavily influence one another which may also confound the results. And finally, due to the characteristics of Polish society, only patients of Caucasian ethnicity were included and our findings may have not be applicable for persons from other ethnic groups.

This study has also several strengths. One of them is its case–control design with strictly matched pairs of case subjects and their comparators, with each pair taken from the very same center. The study was based on a high-quality data sources using samples with a long follow-up time (mean time from diabetes diagnosis to index time has exceeded 10 years) and extensive covariate information, including the date of the onset of diabetes, date of cancer diagnosis, treatment details, metabolic control and other common risk factors, which allowed to explore the relationship between T2DM and cancer risk.

## Conclusions

The elderly obese patients with long-standing and poorly controlled type 2 diabetes treated with high doses of insulin are at high risk of cancer development and they should be rigorously assessed towards malignancies, particularly breast cancer in women and colorectal cancer in men.

The results of our study indicate also that metformin therapy should be implemented in all patients without contraindications and without intolerance to this drug. Insulin in type 2 diabetic patients should be introduced with caution and, if possible, high doses of insulin should be avoided. In addition, strong oncological vigilance should be maintained during the first 10 years of insulin treatment. On the other hand, also deterioration of metabolic control should be avoided and increase of HbA_1c_ level above the threshold of 8.5 % should not be allowed.

Finally, our study indicated the important role of weight management in patients with type 2 diabetes. Thus, it is strongly reasonable to strive for weight reduction in obese diabetic patients to reduce risk of obesity-related complications, including malignancy.

## Abbreviations

AGEs: Advanced glycation end products
AMPK: AMP-activated protein kinase
BMI: Body mass index
CI: Confidence interval
DPP-4: Dipeptidyl-peptidase-4
HbA_1c_: Hemoglobin A_1c_
IGF-1: Insulin-like growth factor-1
LKB1: Liver kinase B1
mTOR: mammalian target of rapamycin
OR: Odds ratio
RAGE: Receptor for AGE
ROS: Reactive oxygen species
SGLT-2: Sodium-glucose transporter
SU: Sulfonylurea
T2DM: Type 2 diabetes mellitus
